# Development and Validation of a Prognostic Nomogram for Colorectal Cancer Patients With Synchronous Peritoneal Metastasis

**DOI:** 10.3389/fonc.2021.615321

**Published:** 2021-07-01

**Authors:** Zifeng Yang, Yong Li, Xiusen Qin, Zejian Lv, Huaiming Wang, Deqing Wu, Zixu Yuan, Hui Wang

**Affiliations:** ^1^ Department of Colorectal Surgery, The Sixth Affiliated Hospital of Sun Yat-Sen University, Guangzhou, China; ^2^ Guangdong Institute of Gastroenterology, Guangdong Provincial Key Laboratory of Colorectal and Pelvic Floor Diseases, Supported by National Key Clinical Discipline, The Sixth Affiliated Hospital of Sun Yat-Sen University, Guangzhou, China; ^3^ Department of General Surgery, Guangdong Provincial People’s Hospital, Guangdong Academy of Medical Sciences, Guangzhou, China

**Keywords:** colorectal cancer, nomogram, prognosis, peritoneal metastasis (PM), synchronous peritoneal metastasis

## Abstract

**Purpose:**

Synchronous peritoneal metastasis (S-PM) is considered a poor prognostic factor for colorectal cancer (CRC) and there is no nomogram to predict the survival of these patients. In this study, we aimed to use a multicenter data to identify the factors associated with S-PM of CRC to construct a nomogram for predicting the overall survival (OS) of these patients.

**Methods:**

CRC patients with S-PM from two medical centers were enrolled between September 2007 and June 2017. Multivariate analysis was used to identify independent factors associated with OS for the nomogram to predict the 1-, 2-, and 3-year OS rates in the development group. The concordance index (C-index), calibration plot, relative operating characteristic (ROC) curve with area under the curve (AUC) were calculated to evaluate the performance of the nomogram in both the development and an external validation group.

**Results:**

277 CRC patients with S-PM in the development group and 68 patients in the validation group were eligible for this study. In multivariate analysis of development group, age, carbohydrate antigen 19-9 (CA19-9), carbohydrate antigen 125 (CA125), cytoreductive surgery (CRS), hyperthermic intraperitoneal chemotherapy (HIPEC), and chemotherapy were independent variables for OS, based on which the nomogram was built. The C-index of the nomogram in the development and validation group was 0.701 (95% Cl, 0.666–0.736) and 0.716 (95% Cl, 0.622–0.810); demonstrating good discriminative ability. The calibration plots showed satisfactory consistency between actual observation and nomogram-predicted OS probabilities in the development and external validation group. The nomogram showed good predictive accuracy for 1-, 2-, and 3-year OS rates in both groups with AUC >0.70. An online dynamic webserver was also developed for increasing the ease of the nomogram.

**Conclusions:**

We developed and validated a predictive nomogram with good discriminative and high accuracy to predict the OS in CRC patients with S-PM.

## Introduction

Colorectal cancer (CRC) is the third most common malignant tumor worldwide, with 1.8 million cases and 881,000 deaths registered globally, in 2018 (1). It is ranked third in morbidity and fifth in mortality in China (2, 3). Currently, radical resection combined with neo-/adjuvant chemotherapy, radiotherapy, or targeted therapy has been shown to be associated with a promising 5-year OS rate of >70% in non-metastatic CRC, and >90% for early CRC (4).

The peritoneum is the third most frequent site for metastasis in CRC, secondary to the liver and lung (5, 6). In regard to synchronous metastatic CRC, the peritoneum is the second most common metastatic site, secondary to the liver (7). Peritoneal metastasis (PM) is associated with poorer progression-free survival and OS, as compared to other CRC metastatic sites (8–10). In the 8th edition of the American Joint Committee on Cancer (AJCC) TNM Classification for CRC, patients with PM are separately classified into an M1c group since they were found to have the worst prognosis compared to patients in the M1a (metastases to one organ) and M1b (metastases to more than one organ) groups (11, 12). At initial diagnosis, 1–13% of CRC patients often present with synchronous peritoneal metastasis (S-PM) (12–14). The prognosis of S-PM has been found to be poorer than metachronous PM (15–17). Once S-PM develops, without active treatment, the patients’ median OS can range between 4 and 7 months (18–21).

According to the Japanese Society for Cancer of the Colon and Rectum (JSCCR) (22) and the National Comprehensive Cancer Network (NCCN) guidelines (23) for the treatment of CRC patients with PM, if complete cytoreduction can be achieved, resection of the isolated peritoneal lesion could be recommended but is advisable to be performed in an experienced cancer center. For incomplete cytoreductive surgery (CRS), the combination of HIPEC with systematic chemotherapy could improve the patient’s survival (24, 25).

Clinically, clinicians need to comprehensively evaluate the imaging findings of PM, tumor marker levels, surgical skill level, development of treatment platform, patient’s symptoms, nutrition condition, patient’s willingness and financial situation, and multiple disciplinary team (MDT) advices, then decide whether to recommend CRS, HIPEC, or palliative chemotherapy. As there is no standard tools to weigh the benefits of these factors for an individualized treatment approach, oncologists can only rely on their clinical experience and judgment; possibly leading to a certain level of bias in selecting treatment methods. Thus, in this study, we aimed to develop and validate a nomogram able to predict the survival of S-PM CRC patients as a tool to help oncologists to make better treatment selection decisions.

## Materials and Methods

### Patients and Study Criteria

S-PM was defined as PM which was concurrently identified at the time of initial primary CRC diagnosis (23). The inclusion criteria for patient selection were: (1) a pathological diagnosis of CRC with S-PM between September 2007 and June 2017; (2) no history of other primary malignant tumors; (3) had complete clinical and follow-up data. Patients were excluded if the clinicopathological information was incomplete or died from other diseases. Clinicopathological parameters included sex, age, body mass index (BMI), carcinoembryonic antigen (CEA), CA19-9, CA125, computed tomography (CT) findings of PM, other organ-invasion, other metastasis, tumor location, digestive obstruction, fistulation or bypass, CRS, HIPEC, chemotherapy, and differentiation grade. The patients were classified in a development group, comprising of patients from The Sixth Affiliated Hospital of Sun Yat-sen University (Guangzhou, China), and a validation group, which comprised of patients from the Guangdong Provincial People’s Hospital (Guangzhou, China).

### Treatment Approaches: CRS, HIPEC, and Chemotherapy

In case that the tumor burden was deemed resectable or caused severe perforation, bleeding or obstruction, CRS was performed using primary tumor removal, invaded-organ resection, and/or peritonectomy techniques. The degree of CRS was evaluated by the completeness of cytoreduction score (CCR score) after surgery. CCR0 was assigned for no remaining visible cancer lesion after the CRS. CCR1, 2, and 3 were assigned if the remaining lesions were less than 2.5 mm, 2.5 to 2.5 cm, and greater than 2.5 cm, respectively.

HIPEC was conducted using the closed abdomen technique after surgery. Briefly, four tubes (two for the inflow of chemotherapy reagents and saline solution at 42 °C and two for outflow liquid) were inserted into the abdomen. Several HIPEC chemotherapy regimens (i.e. 5-fluorouracil, Cisplatin, 5-fluorouracil plus Cisplatin, Paclitaxel, Oxaliplatin, 5-fluorouracil plus Lobaplatin) were used. The duration of each HIPEC treatment was at least 1 h. In addition, all cases underwent at least two courses of HIPEC during the first 24–72 h after CRS.

Chemotherapy included perioperative chemotherapy (neo- and adjuvant chemotherapy) or palliative chemotherapy. In some cases, targeted therapy was added. The chemotherapy regimens were 5-fluorouracil based chemotherapy (i.e. FOLFOX, FOLFIRI, or XELOX). Targeted therapy contained Cetuximab or Bevacizumab. All patients with chemotherapy received at least four courses of continuous therapy.

### Follow-Up, Univariate and Multivariate Analysis

The last follow-up time of all the patients was on May 2019 or the date of registered death prior to May 2019. The endpoint of this study was OS, calculated from the date of initial biopsy diagnosis to death. Survival curves were drawn using the Kaplan–Meier plots. Univariate analyses were conducted to identify prognostic factors associated with OS in the development group. Factors with a *P <*0.05 in the univariate analysis were selected for multivariate Cox regression analysis.

### Development and Validation of the Nomogram

Independent variables from multivariate analyses with a *P <*0.05 were used to develop a nomogram able to predict the 1-, 2-and 3-year OS rate of CRC patients with S-PM. To decrease the risk of bias, an internal validation using the development group and an external validation using the validation group were performed. The interpretation of the probability of C-index between predicted and actual outcome was used to evaluate the predictive ability and discriminative ability of the nomogram model of the development and validation groups. The value of the C-index should be 0.5–1.0. 0.5 of C-index to indicate random chance, and 1.0 indicated a perfect discriminative ability. The fitting degree of the nomogram was assessed in the development and validation groups using calibration plots. The Relative Operating Characteristic (ROC) curve with the area under the curve (AUC) was used to evaluate the discriminative and predictive ability in both groups. A user-friendly webserver was then built based on the validated nomogram to facilitate the use of the nomogram.

### Statistical Analysis

Statistical analyses were performed using the R (www.R-project.org, version 3.6.3), SPSS (version 22.0 for Windows; SPSS, Chicago, IL, USA), and GraphPad Prism (version 8.2.1) software. The R statistical packages “rms”, “survival”, “foreign”, “survivalROC”, “DynNom”, “shiny”, and “rsconnect” were used to calculate the C-index, plot the calibration and ROC curve, construct the nomogram and build the webserver. Chi-squared test, Kaplan–Meier plot, univariate, and multivariate Cox regression analysis were calculated by using SPSS software. The Forest plot was drawn by GraphPad Prism software. A *p* value <0.05 was considered statistically significant.

## Results

### Patients and OS

**Figure 1:**
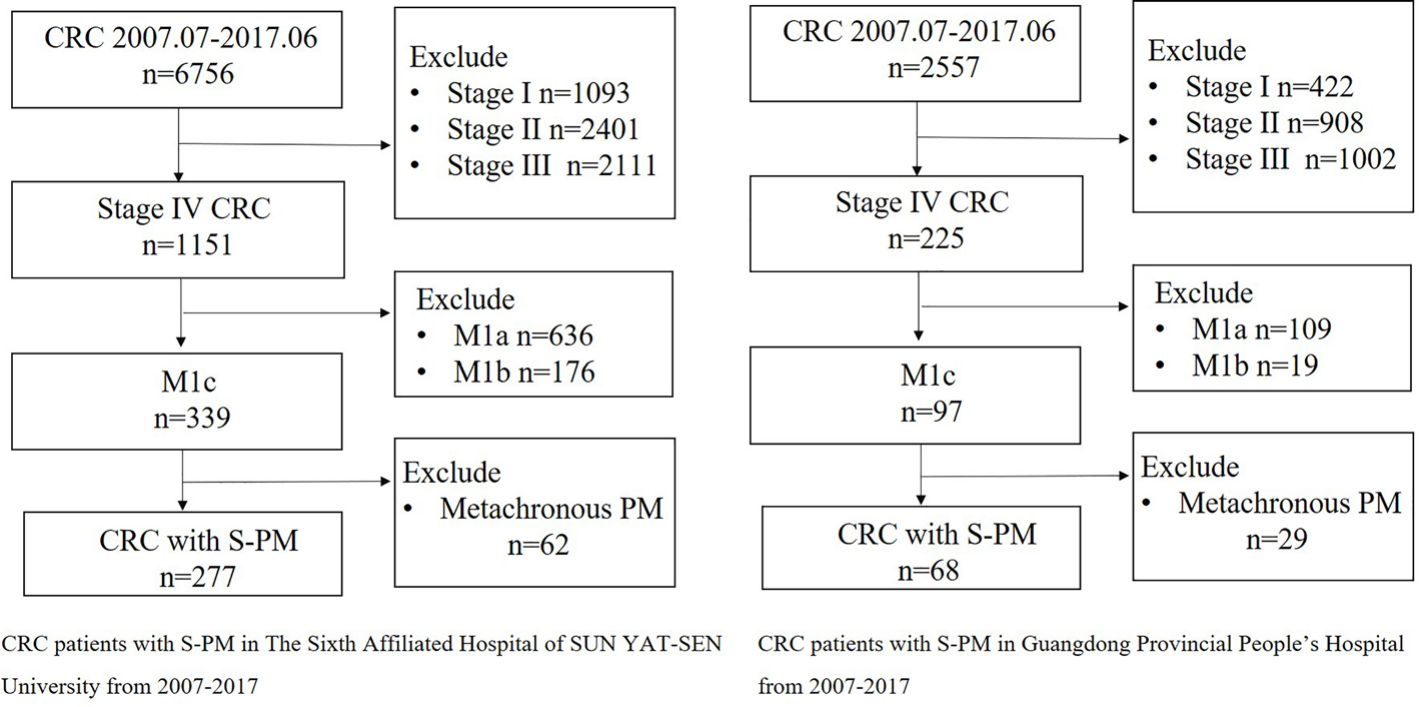
Flow chart of patient selection in two medical centers.

A total of 345 patients, 277 patients from the development group and 68 from the validation group, with pathologically diagnosed CRC with S-PM were included in the study **(** **Figure 1** **)**. The clinicopathological factors and the therapeutic details of the patients are shown in **Table 1** and **Table S1**. Among the 277 patients from the development group, 198 patients (71.5%) received CRS. Of them, 54 (19.5%) patients were classified as CCR0-1 and 144 (52.0%) as CCR2-3. Further, 61 patients (22.0%) received HIPEC and 149 patients (53.8%) received chemotherapy.

**Table 1:** A part of characteristics of CRC patients with S-PM in the development and validation groups.

Variables	All patients	Development group	Validation group	*p*-value
	N = 345	N = 277	N = 68	
**Age (years)**				0.762
≤65	253 (73.3%)	204 (73.6%)	49 (72.1%)	
>65	92 (26.7%)	73 (26.4%)	19 (27.9%)	
**CA19-9 (u/ml)**				0.684
≤37	191 (55.4%)	155 (56.0%)	36 (52.9%)	
>37	154 (44.6%)	122 (44.0%)	32 (47.1%)	
**CA125 (U/ml)**				0.412
≤35	143 (41.4%)	118 (42.6%)	25 (36.8%)	
>35	202 (58.6%)	159 (57.4%)	43 (63.2%)	
**Fistulation or bypass**				0.625
No	270 (78.3%)	215 (77.6%)	55 (80.9%)	
Yes	75 (21.7%)	62 (22.4%)	13 (19.1%)	
**Other metastasis**				0.207
Absent	221 (64.1%)	182 (65.7%)	39 (57.4%)	
Present	124 (35.9%)	95 (34.3%)	29 (42.6%)	
**CRS**				0.117
No	99 (28.7%)	79 (28.5%)	20 (29.4%)	
CCR 0-1	94 (27.2%)	54 (19.5%)	12 (17.6%)	
CCR 2-3	152 (44.1%)	144 (52.0%)	36 (53.0%)	
**HIPEC**				0.001
No	246 (71.3%)	216 (78.0%)	30 (44.1%)	
Yes	99 (28.7%)	61 (22.0%)	38 (55.9%)	
**Chemotherapy**				0.006
No	147 (42.6%)	128 (46.2%)	19 (27.9%)	
Yes	198 (57.4%)	149 (53.8%)	49 (72.1%)	

CA19-9, carbohydrate antigen 19-9; CA125, carbohydrate antigen 125; CRS, cytoreductive surgery; CCR, completeness of cytoreduction; HIPEC, hyperthermic intraperitoneal chemotherapy.

In the validation group, 48 patients (70.6%) received CRS, of whom 12 (17.6%) and 36 (53.0%) patients were classified as CCR0-1 and CCR2-3, respectively. Also, 38 patients (55.9%) received HIPEC and 49 patients (72.1%) received chemotherapy.

Significant differences in terms of CT findings (*p =* 0.001), other organ-invasion (*p <*0.001), digestive obstruction (*p <*0.001), HIPEC (*p =* 0.001) and chemotherapy (*p =* 0.006) were observed between the development and validation groups (**Table 1** and **Table S1**). The mean OS for all patients was 16 (1–119) months and the 1-, 2-, and 3-year OS rates were 59.8, 37.7, and 27.0%, respectively. In the development group, the 1-, 2-, and 3-year OS rates were 54.9, 33.2, and 23.1%, respectively, and in the validation group, they were 66.7, 44.5, and 33.3%, respectively.

### Univariate and Multivariate Cox Regression Analyses in the Development Group

For the development group, univariate analyses identified age ≤65 years (*p <*0.001), CA19-9 ≤37 u/ml (*p <*0.001), CA125 ≤35 U/ml (*p <*0.001), absence of fistulation or bypass (*p <*0.001), absence of distant metastasis (*p* = 0.015), CRS (*p <*0.001), HIPEC (*p* = 0.001) and chemotherapy (*p* = 0.004) as factors that were associated with better prognosis in CRC patients with S-PM (**Table 2**). Of them, age, CA19-9, CA125, CRS, HIPEC, and chemotherapy were found to be independent prognostic factors for OS in multivariate analyses (**Table 2**) and were therefore used for building the nomogram.

**Table 2:** Univariate and multivariate analyses of the 277 CRC patients with S-PM in the development group.

Variables	Univariate analysis		Multivariate analysis	
	HR (95%CI)	*P*-value	HR (95%CI)	*P*-value
**Age (≤65 years)**	1.766 (1.326–2.352)	<0.001	1.445 (1.070–1.951)	0.016
**CA19-9 (u/ml) (≤37)**	1.663 (1.279–2.163)	<0.001	1.447 (1.085–1.929)	0.012
**CA125 (U/ml) (≤35)**	1.658 (1.268–2.168)	<0.001	1.375 (1.040–1.819)	0.026
**Fistulation or bypass (No)**	2.269 (1.673–3.077)	<0.001	1.206 (0.794–1.898)	0.394
**Other metastasis (Absent)**	1.397 (1.067–1.749)	0.015	1.098 (0.851–1.518)	0.529
CCR0-1	0.277 (0.184–0.419)	<0.001	0.407 (0.241–0.685)	0.001
CCR2-3	0.486 (0.362–0.653)	<0.001	0.613 (0.422–0.934)	0.023
**HIPEC (No)**	0.561 (0.398–0.790)	0.001	0.659 (0.464–0.935)	0.020
**Chemotherapy (No)**	0.680 (0.523–0.883)	0.004	0.702 (0.537–0.919)	0.010

CA19-9, carbohydrate antigen 19-9; CA125, carbohydrate antigen 125; PM, peritoneal metastasis; CRS, primary tumor resection; HIPEC, hyperthermic intraperitoneal chemotherapy.

### Construction and Validation of the Nomogram

**Figure 2:**
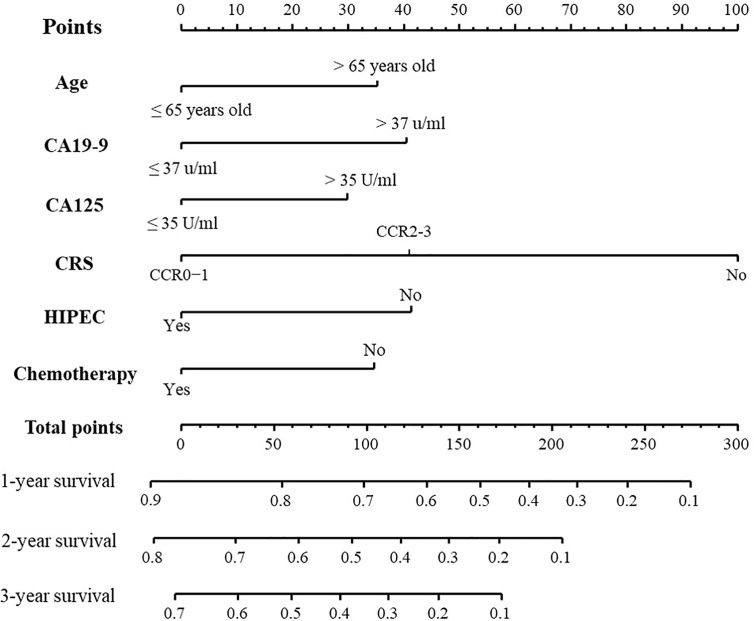
Nomogram for predicting the OS of CRC patients with S-PM. The C-index of this nomogram is 0.701 (95% Cl, 0.666–0.736).

The forest plot and survival curves of six independent factors were shown in **Figure S1**. The 1-, 2-, 3-year survival-predicting nomograms in the development group were presented in **Figure 2**.

**Figure 3:**
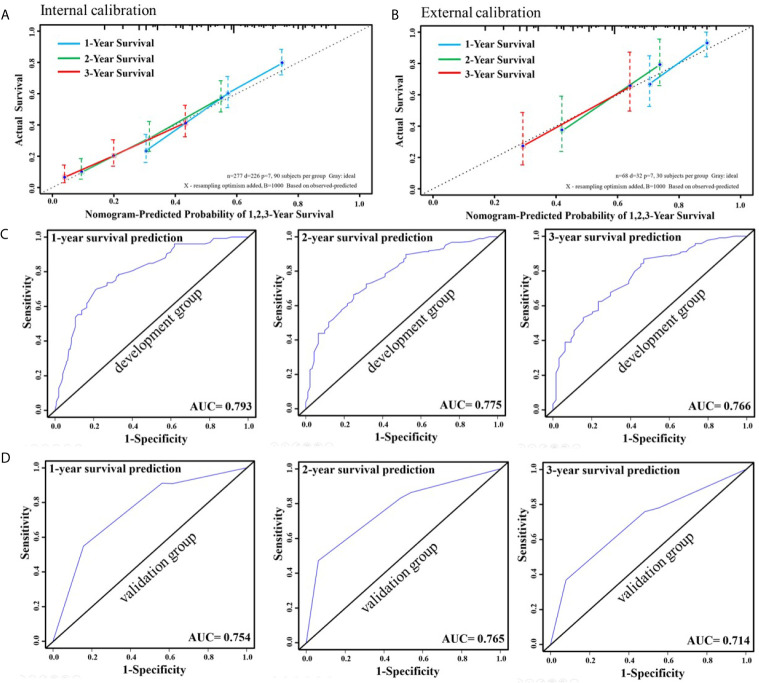
Internal calibration curve to validate nomogram model for 1-, 2-, and 3-year survival and its C-index was 0.701 (95% Cl, 0.666–0.736) **(A)**. External calibration curve to validate nomogram model for 1-, 2-, and 3-year survival and its C-index was 0.716 (95% Cl, 0.622–0.810) **(B)**. ROC curve of 1-, 2-, and 3-year survival prediction in the development group **(C)**. ROC curve of 1-, 2-, and 3-year survival prediction in the validation group **(D)**.

As shown in **Figure 3A**, in the internal validation cohort (development group), the C-index for the nomogram to predict the OS of CRC patients with S-PM was 0.701 (95% Cl, 0.666–0.736). For the calibration plot, the dotted line represents the predicted values of the nomogram, while the colorful line represents the actual values of the 1-, 2-, and 3-year OS rates. The less discrepant they are, the more precise the predictive capability of the nomogram was. In the external validation cohort (validation group), the C-index was 0.716 (95% Cl, 0.622–0.810). This was higher than the development group, which indicated the nomogram model obtained an ideal predictive accuracy. The external calibration plot for the nomogram showed good agreement between the predicted and actual survival rates (**Figure 3B**).

For the internal calibration, the colorful lines fluctuated above and below the dotted line, to identify a reliable predictive capability of the nomogram. The AUC of 1-, 2-, and 3-year OS predictions of the development group were 0.793, 0.775, and 0.766, respectively. These results indicated favorable discrimination of this proposed nomogram (**Figure 3C**). The AUC of 1-, 2-, and 3-year survival predictions of the validation group were 0.754, 0.765, and 0.714 (**Figure 3D**). Favorable discrimination was shown and the results were very close to that of the development group.

### Implementation of the Webserver

**Figure 4:**
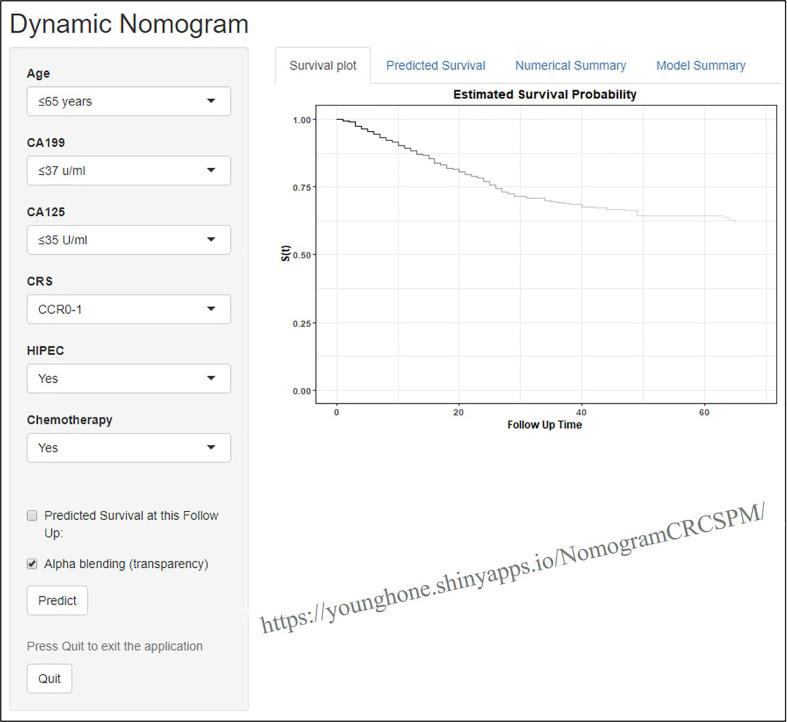
Webserver display of the online dynamic nomogram.

An online dynamic platform (https://younghone.shinyapps.io/NomogramCRCSPM/) was developed to increase the applicability of the proposed nomogram **(** **Figure 4** **)**. It can assist researchers and clinicians to more easily obtain the survival probability of their patients by inputting their corresponding clinical factors, after which the webserver will generate the output read in the forms of figures and tables.

## Discussion

In this study, the incidence of CRC with S-PM was found to be 4.1% (277/6,756) in the development group and 2.7% in the validation group; within the range of 1–13% in previous studies (14, 19, 26). The primary tumor was mainly located in the right side of the colon (44.3%, 153/345). Left-sided (34.2%, 118/345) and rectal tumors accounted for only 21.4% (74/345), which was similar to some previous studies (13, 19, 27). The rate of S-PM with distant metastases (liver or lung are the most common site) was 36.2–74.1% in previous studies (11, 13, 26, 27), while it was 35.9% (124/345) in our study. The diagnosis of CRC with S-PM was often made at an advanced stage due to the lack of specific symptoms of peritoneal involvement and the low sensitivity of current imaging techniques in detecting PM (14, 27). In this study, only 41.4% (143/345) could be diagnosed by contrast-enhanced CT scans, so the diagnostic technology still needs to be improved.

Laparoscopic exploration or laparotomy is considered the gold standard for the diagnosis of PM (28). In this study, there were significant differences in imaging diagnosis, other organ-invasion, digestive obstruction, HIPEC, and chemotherapy between the development group and the validation group (p <0.05). This might be related to the degree of peritoneal invasion, the local invasion of the primary tumor, the differences in diagnostic criteria, therapeutic level, and the treatment concept of the MDT in different medical centers (14, 17). The 1-, 2-, and 3-year OS rates were different in the two groups, possibly due to more cases in the validation group who underwent HIPEC and chemotherapy, as HIPEC and chemotherapy were identified as independent factors for good survival. Then, we performed univariate analysis in the development group and identified prognostic factors of survival including age, the levels of CA19-9 and CA125, fistulation or bypass, distant metastasis, CRS, chemotherapy, and HIPEC. The elderly patients were usually presented with a poor prognosis with poor physical function, lower immune function, and lack of sufficient treatment (29–31). Furthermore, we built a nomogram to predict prognosis for CRC patients with S-PM, which may be used to guide clinical practice.

Previous studies have shown that CA19-9 and CA125 were independent factors of prognosis in CRC patients with PM (32, 33). In this study, the overall positive rate (>37 u/ml) of CA19-9 was 49.5%, compared to 45.6–62.7% in previous studies (27). Meanwhile, CA19-9 was an independent prognostic factor and could be used during surveillance for early detection of disease recurrence or aggravation (24, 31, 33, 34). CA125 was identified as a sensitive tumor marker for ovarian tumors. However, a recent study on 853 patients demonstrated that CA125 could be more significant in predicting the prognosis of PM in CRC in both males and females than CEA (35). Huo (33) and Chuk (21) also found that CA125 was an independent risk factor of prognosis and could be used as a prognostic predictor.

Current guidelines and related studies confirm that CRS, HIPEC, and systematic chemotherapy in selected CRC with S-PM cases could significantly improve the long-term survival of these patients (17, 22, 36). However, to comprehensively evaluate and consider the patients’ physical function, nutrition level, willingness, economic situation, tumor marker value, surgical skill level, development of treatment platform, and multiple disciplinary team (MDT) discussion results in the busy daily clinical practice is quite laborious and could vary from clinicians to clinicians (14). Therefore, not all CRC patients with S-PM have the opportunity to simultaneously undergo CRS, HIPEC, and systematic chemotherapy. In our study, 62.8% (199/317) of CRC patients with S-PM underwent CRS (CCR0-3), while in Wang (27) and Tanaka’s (12) study, the proportion of CRS was 45.6 and 88.4%. Previous literature reports showed that the HIPEC treatment rates of CRC patients with S-PM were 21% (27) and 73% (37), while only 38.2% (121/317) CRC patients with S-PM received HIPEC treatment in our study. Further, in this study, a total of 54.9% (174/317) of the patients underwent perioperative chemotherapy, compared to 53.3–70.1% in other studies (10, 11, 16, 27). Meanwhile, as a relatively mature and effective treatment, previous studies have shown that high-quality CRS, standard HIPEC treatment and systematic perioperative chemotherapy could improve the prognosis of CRC patients with PM (14, 23). It is important to note that, for selective PM cases, on the basis of CRS combined with HIPEC, standard perioperative chemotherapy could better improve the prognosis (22, 23).

Complete CRS plus HIPEC, and systematic perioperative chemotherapy can improve the prognosis of CRC with S-PM (13, 22, 25). HIPEC comprises of intraperitoneal perfusion of chemotherapy reagents, heated to 42 °C to eliminate microscopic disease. The HIPEC technique is currently controversial for drug regimens, volume of infusion, duration, and concentrations in S-PM (38). A study from Australia showed that oxaliplatin offers a survival advantage when used for HIPEC in CRC with PM (39). As for stage IV CRC patients, whether or not it requires CRS, the NCCN guidelines (22) and relevant studies (40, 41) recommend 5-fluorouracil or capecitabine-based systemic chemotherapy to improve the prognosis. In this study, the HIPEC and chemotherapy were independent factors affecting the prognosis.

Although several nomograms had been developed to predict the OS for PM or stage IV CRC (8, 29, 42), no nomogram for predicting the OS of CRC with S-PM has yet been reported. In this study, an OS-predicting nomogram for S-PM was established and had a promising C-index of 0.701, signifying decent discriminatory ability of the nomogram. We used an independent cohort from other medical center for external validation and similarly observed a promising C-index of 0.716, further validating the good predictive performance of this proposed nomogram. Further, the calibration plot for 1-, 2-, and 3-year survival showed a first-rank consistency between the predicted and actual observation in both the development and validation group; indicating the reproducibility and reliability of this nomogram. Next, to make this nomogram easy to use in clinical practice, we developed an online time-saving dynamic nomogram, https://younghone.shinyapps.io/NomogramCRCSPM/, which can output a prognostic predictive value by inputting corresponding indicators.

Despite the interesting findings showed in this study, there were several potential limitations worth mentioning. First, this was a retrospective study and the cohort size could be considered limited; thus, potential selection bias might have existed. Second, the details of the peritoneal cancer index (PCI), the most widely used index to predict the survival of patients with PM (16, 17, 43), was unavailable due to the retrospective nature or incomplete data, and was thereby not calculated in this study. Third, the developed and validated data came from different medical centers and might differ in treatment concepts and details, and the number of cases in the external verification group is small. Therefore, we plan to conduct a prospective trial to validate our nomogram and its applicability in the clinic in the future.

## Conclusions

We constructed and validated a nomogram able to predict the 1-, 2-, and 3-year OS for CRC patients with S-PM with good discriminative and high accuracy. The proposed nomogram could be used as a tool for more accurate prediction of individual prognosis and improve oncologists’ clinical decision-making when formulating personalized treatments of CRC patients with S-PM.

## Data Availability Statement

The raw data supporting the conclusions of this article will be made available by the authors, without undue reservation.

## Ethics Statement

The studies involving human participants were reviewed and approved by The Ethics Committees of The Sixth Affiliated Hospital (No. 2020ZSLYEC-109). Written informed consent for participation was not required for this study in accordance with the national legislation and the institutional requirements. Written informed consent was not obtained from the individual(s) for the publication of any potentially identifiable images or data included in this article.

## Author Contributions

ZfY, DqW, YL, and HW contributed to the idea and design. ZfY, XsQ, ZxY, HmW, and ZjL contributed to the data acquisition and analysis. ZfY, DyW, XsQ, and ZxY contributed to the manuscript writing and revision. All authors contributed to the article and approved the submitted version.

## Conflict of Interest

The authors declare that the research was conducted in the absence of any commercial or financial relationships that could be construed as a potential conflict of interest.

## Supplementary Material

Supplementary Figure 1 Forest plot showed multivariate analyses of OS in the development group **(A)**. OS curve of age, CA19-9, CA125, CRS, HIPEC and chemotherapy in the development group **(B)**.
