# Supplementary figures and images for: Development and Validation of a Prognostic Nomogram for Colorectal Cancer Patients With Synchronous Peritoneal Metastasis
Source: Front Oncol. 2021 Jul 1;11:615321. doi: 10.3389/fonc.2021.615321 (PMC8281961; doi:10.3389/fonc.2021.615321)

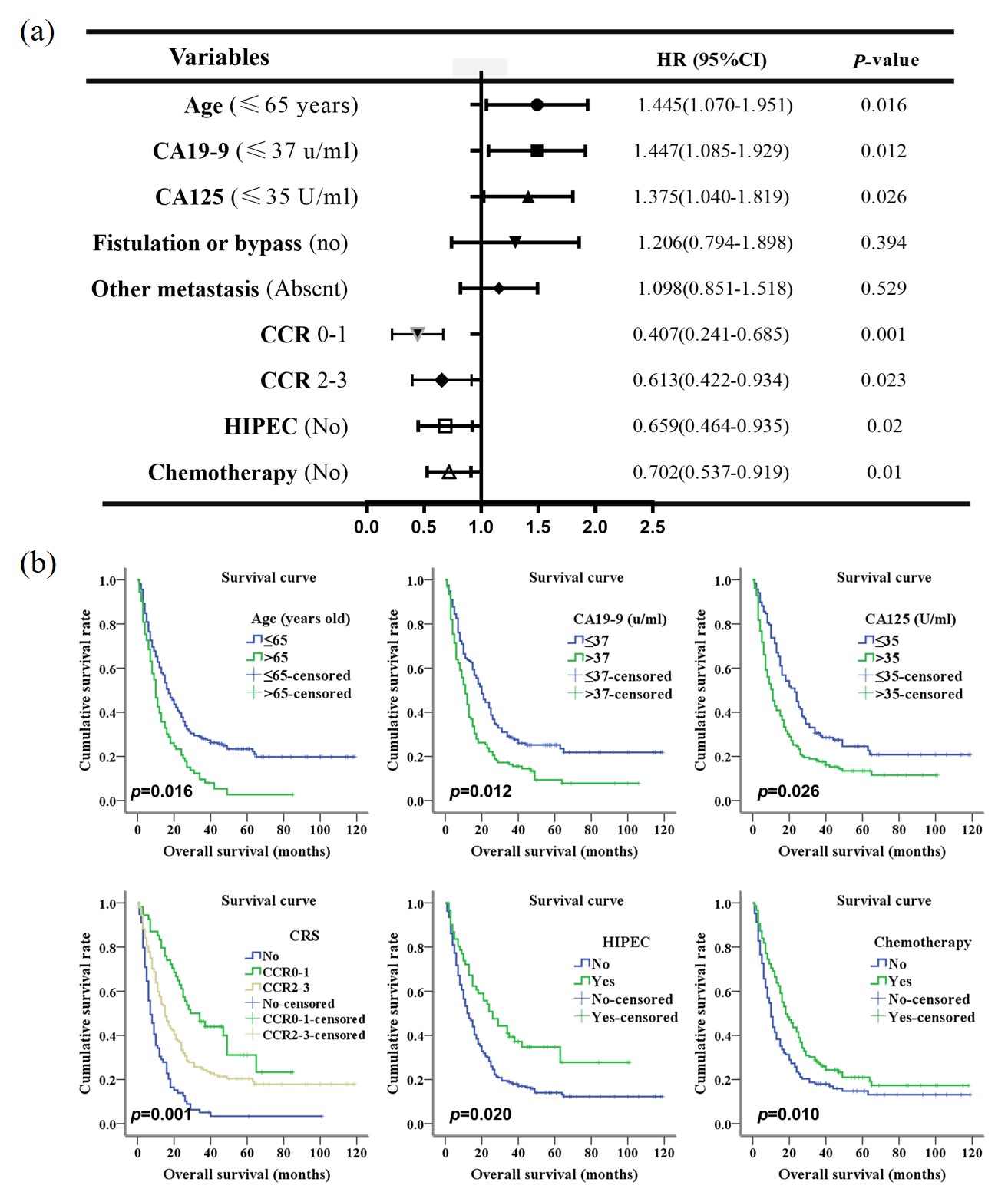

Supplement: Supplementary Figure 1 — Forest plot showed multivariate analyses of OS in the development group (A). OS curve of age, CA19-9, CA125, CRS, HIPEC and chemotherapy in the development group (B). [file Image_1.jpeg]
