# Supplementary material for: Development and Validation of a Prognostic Nomogram for Colorectal Cancer Patients With Synchronous Peritoneal Metastasis
Source: Front Oncol. 2021 Jul 1;11:615321. doi: 10.3389/fonc.2021.615321 (PMC8281961; doi:10.3389/fonc.2021.615321)
Supplement: Supplementary file 2 [file Table_1.docx]

**Table S1** The other characteristics of CRC patients with S-PM in the development and validation groups.

| **Variables** | **All patients**  **N=345** | **Development group**  **N=277** | | **Validation group**  **N=68** | ***p*-value** |
| --- | --- | --- | --- | --- | --- |
| **Sex** |  |  |  | | 0.782 |
| Male | 210 (60.9%) | 170 (61.4%) | 40 (58.8%) | |  |
| Female | 135 (39.1%) | 107 (38.6%) | 28 (41.2%) | |  |
| **BMI（kg/m^2^）** |  |  |  | | 1.000 |
| ≤ 18 | 76 (22.0%) | 61 (22.0%) | 15 (22.1%) | |  |
| ＞18 | 269 (78.0%) | 216 (78.0%) | 53 (77.9%) | |  |
| **CEA (ng/ml)** |  |  |  | | 0.776 |
| ≤ 10 | 175 (50.7%) | 142 (51.3%) | 33 (48.5%) | |  |
| ＞10 | 170 (49.3%) | 135 (48.7%) | 35 (51.5%) | |  |
| **CT findings of PM** |  |  |  | | 0.001 |
| No | 202 (58.6%) | 150 (54.2%) | 52 (76.5%) | |  |
| Yes | 143 (41.4%) | 127 (45.8%) | 16 (23.5%) | |  |
| **Other organ-invasion** |  |  |  | | <0.001 |
| Absent | 110 (31.9%) | 63 (22.7%) | 47 (69.1%) | |  |
| Present | 235 (68.1%) | 214 (77.3%) | 21 (30.9%) | |  |
| **Tumor location** |  |  |  | | 0.201 |
| Right side | 153 (44.3%) | 119 (43.0%) | 34 (50.0%) | |  |
| Left side | 118 (34.2%) | 101 (36.5%) | 17 (25.0%) | |  |
| Rectum | 74 (21.4%) | 57 (20.5%) | 17 (25.0%) | |  |
| **Digestive obstruction** |  |  |  | | <0.001 |
| Absent | 162 (47.0%) | 108 (39.0%) | 54 (79.4%) | |  |
| Present | 183 (53.0%) | 169 (61.0%) | 14 (20.6%) | |  |
| **Differentiation grade** |  |  |  | | 0.051 |
| Poorly or undifferentiated | 131 (38.0%) | 98 (35.4%) | 33 (48.5%) | |  |
| Moderated or high | 214 (62.0%) | 179 (64.6%) | 35 (51.5%) | |  |

**BMI**, Body Mass Index. **CEA**, carcinoembryonic antigen. **CT**, computed tomography. **PM**, peritoneal metastasis**.**
